# The feasibility, time savings and economic impact of a designated time appointment system at a busy HIV care clinic in Kenya: a randomized controlled trial

**DOI:** 10.7448/IAS.18.1.19876

**Published:** 2015-07-09

**Authors:** Zachary A Kwena, Betty W Njoroge, Craig R Cohen, Patrick Oyaro, Rosemary Shikari, Charles K Kibaara, Elizabeth A Bukusi

**Affiliations:** 1 Center for Microbiology Research, Kenya Medical Research Institute, Kisumu, Kenya; 2 Department of Obstetrics, Gynaecology & Reproductive Sciences, University of California at San Francisco, San Francisco, CA, USA

**Keywords:** HIV patient, designated time appointment system, HIV care clinic, Kenya, clinic waiting time

## Abstract

**Introduction:**

As efforts are made to reach universal access to ART in Kenya, the problem of congestion at HIV care clinics is likely to worsen. We evaluated the feasibility and the economic benefits of a designated time appointment system as a solution to decongest HIV care clinics.

**Methods:**

This was an explanatory two-arm open-label randomized controlled trial that enrolled 354 consenting participants during their normal clinic days and followed-up at subsequent clinic appointments for up to nine months. Intervention arm participants were given specific dates and times to arrive at the clinic for their next appointment while those in the control arm were only given the date and had the discretion to decide on the time to arrive as is the standard practice. At follow-up visits, we recorded arrival and departure times and asked the monetary value of work participants engaged in before and after clinic. We conducted multiple imputation to replace missing data in our primary outcome variables to allow for intention-to-treat analysis; and analyzed the data using Mann–Whitney *U* test.

**Results:**

Overall, 72.1% of the intervention participants arrived on time, 13.3% arrived ahead of time and 14.6% arrived past scheduled time. Intervention arm participants spent a median of 65 [interquartile range (IQR), 52–87] minutes at the clinic compared to 197 (IQR, 173–225) minutes for control participants (*p*<0.01). Furthermore, intervention arm participants were more productively engaged on their clinic days valuing their cumulative work at a median of USD 10.5 (IQR, 60.0–16.8) compared to participants enrolled in the control arm who valued their work at USD 8.3 (IQR, 5.5–12.9; *p*=0.02).

**Conclusions:**

A designated time appointment system is feasible and provides substantial time savings associated with greater economic productivity for HIV patients attending a busy HIV care clinic.

## Introduction

Currently, over 35 million people are living with HIV worldwide; 67% of whom live in sub-Saharan Africa [1]. The advent of antiretroviral therapy (ART) in managing HIV patients in the mid-1990s transformed an otherwise terminal illness to a chronic disease [2]. Thus, initiating and retaining people on ART have become critical for both prevention and treatment success especially as we embrace early HIV treatment [3–6]. Even with only about 60% of 15 million people eligible in receiving care, healthcare systems in sub-Saharan Africa are already experiencing challenges coping with the large number of patients. These challenges result in long patient waiting times as witnessed in most of HIV care clinics across sub-Saharan Africa [7–10]. This situation is likely to worsen with continued scale-up of ART and care services through local country initiatives as well as international mechanisms such as President's Emergency Plan for AIDS Relief (PEPFAR), the Global Fund and other United Nation (UN) initiatives [11]. Care programmes have tried different interventions including task shifting to improve clinic operations and patient experiences but with modest impact on reducing long waiting times and congestions [12, 13]. With changing guidelines for ART initiation, additional patients will become eligible for ART, increasing potential numbers at the clinics. As more people enrol into care against unmatched expansion of health services, the challenge of long patient waiting times is likely to worsen [14, 15].

Many negative outcomes are associated with long patient waiting times at HIV care clinics [16]. Some of these negative outcomes include missed income, missed school attendance (for children), congestion that may lead to easy spread of infectious diseases, lost opportunity to fulfil socio-economic responsibilities and general service dissatisfaction adding to loss to follow-up [14]. Previous studies that sought to determine ways to minimize patient waiting time mainly focused on patient/client flow analyses to detect the amount of time patients take at each department and how it can be reduced to save on the overall time at the clinics [14, 16, 17]. However, the main problem is the time patients have to wait before they are seen by clinicians. This is because most patients arrive almost at the same time in the morning, and some virtually wait the whole day at the clinic. The alternative is graduating the time patients come to the clinic through a designated time appointment system. This kind of scheduling system may provide opportunities for patients to plan and engage in other gainful activities before and after their clinic visits. Although this system in combination with others may have the potential to reduce waiting time [18, 19], it has not been systematically evaluated in African HIV care settings where the prevailing opinion is that designated time appointment systems are not feasible. Thus, we evaluated the feasibility and the personal economic benefits of a designated time appointment system as a solution to decongest a large HIV care and treatment clinic in Kisumu, Kenya.

## Methods

### Study design

This was an explanatory two-arm open-label randomized controlled trial in which 354 patients were randomly assigned to either a designated time appointment system (intervention) or the standard scheduled patient drop-in system (control); data were collected on three visits up to nine months after enrolment.

### Settings

This study was carried out between March 2012 and July 2013 at the Family AIDS Care and Education Services (FACES) clinic, which is a busy urban HIV care clinic situated at the Lumumba Health Centre, Kisumu, Kenya. Founded in 2004, FACES is an HIV/AIDS care and treatment programme funded by PEPFAR via the Centres for Disease Control and Prevention. The programme uses a family model of care that focuses on identifying and enrolling all HIV infected family members and retaining and supporting them in care. The family model of care is based on the linkage between HIV-infected patients (index patients) and their family members at risk. Providers guide index patients through the steps of identifying family members at HIV risk, address disclosure, facilitate family testing and work to enrol all HIV-positive family members and prevent new infections [20]. By December 2013, the FACES programme at the Lumumba Health Centre had a total of 5464 patients currently enrolled in care out of whom 4289 (78.5%) were on ART (www.faces.org accessed on 18 April 2014). At FACES clinic, and indeed, most other government-supported HIV clinics, stable patients are given an appointment duration of three months. The volume of patients booked for each day differs, but they are allocated equally to all providers present on any particular day as they arrive. This ensures that providers are not overburdened in comparison to others. Typically, patients spent three to four hours at the clinic from arrival to departure.

### Study sample size and endpoints

We based sample size calculation on our primary endpoint, which was the time spent at the clinic in the intervention and control groups. To detect a 30% reduction in the time spent at care clinic with a two-sided 5% significance level and a power of 80%, a sample size of 177 patients per arm was necessary. To recruit this number of patients, a six-month accrual period was anticipated. Our secondary endpoint was the proportion of participants arriving on time, ahead of time and past time. Thus, we obtained the proportion of participants who arrived (a) before their scheduled time, (b) on the scheduled time and (c) after scheduled time. While arriving before and on schedule time amounted to keeping assigned appointment time, it was necessary to separate the categories to monitor their dynamics over time. We also determined how much time they arrived before and after their scheduled times. Our other endpoint was self-reported value of time.

### Study procedures

**Figure 1:**
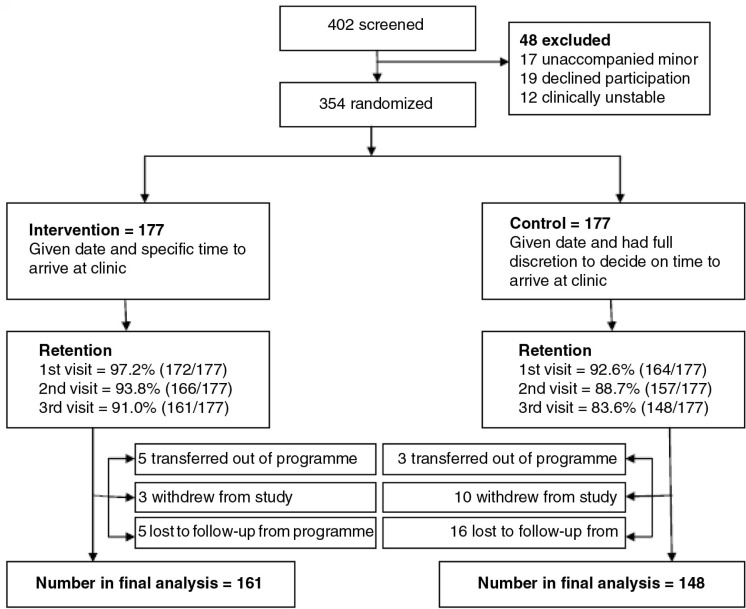
Screening and enrolment flow diagram of participants enrolled in designated time appointment study.

Our study participants were drawn from HIV-positive patients receiving care at the FACES-supported clinic in Lumumba Health Centre, Kisumu, Kenya. Four-hundred and two participants were approached to participate by the study staff at the end of their usual clinic visits. Those interested were referred to the study's private room where they were given additional details, screened for eligibility and consented into the study by the study team (Figure 1). Participants were enrolled into the study if they attended a clinic visit at the FACES-supported clinic at Lumumba Health Centre between March and August 2012 (i.e. during the enrolment dates), were medically stable upon clinical evaluation, patients were at least 18 years of age and, they were able and willing to give written informed consent. Length of time since HIV diagnosis and enrolment in ART were not inclusion criteria. The participants were enrolled during their normal clinic days and followed-up at subsequent clinic appointments. They were asked to commit to a follow-up duration of up to nine months (three clinic appointment visits). The consenting participants completed a brief baseline questionnaire after which they were randomized either to designated appointment time where they were given date and time for the next visit (intervention) or standard scheduled drop-in where they were given next return date but they had full discretion to decide on the time to arrive at the clinic as is the practice at Ministry of Health facilities in Kenya (control). We used biostatistician pre-randomized envelopes numbered sequentially with participants being assigned to the arm indicated in the sealed envelopes in the order in which they were enrolled. We used random number generator to allocate arms to each number in the sequence of 1–354. Research interviewers counselled participants on the importance of keeping their next appointments given by clinicians and the procedure to follow when they arrived at the clinic. Participants arriving for their visits were received at the study office and given a time stamp card that captured their arrival time. The card also captured the time they reported at various benches until they completed their clinic procedures. After completing their clinic procedures, they were referred to the study office where they completed a short follow-up questionnaire. The participants surrendered their time stamp card to the research staff before departure from the clinic. The study was designed to fit into FACES clinic schedules and procedures with minimum interruption. All clinicians at the site shared the day's workload equally and each one of them took care of study participants as part of their normal workflow. Sharing out the day's workload equally among clinicians on duty ensured that control participants were not unfairly disadvantaged. Programme clinicians were immediately available for intervention participants who arrived on time for their scheduled appointments to take them through the routine clinic procedures. The clinicians had 10 minutes between scheduled participants. Intervention participants who arrived early waited for their time slot and those who arrived late, that is, more than five minutes after their scheduled time, were seen at the next available time. On arrival, control participants followed the standard procedure at FACES where they are expected to queue to be served on the first-come-first-served basis.

### Measurements

Data for this study were divided into two sections; (a) socio-demographic and treatment data that were retrieved from archived FACES data and (b) travel, clinic procedures as well as before and after clinic activities data collected through baseline and follow-up questionnaire. At baseline, we collected data about frequency of visits, distance between home and clinic, means of transport available and used, socio-economic activities planned for before and after clinic, time spent at clinic, flow of patients at clinic, ability to keep time and average time spent with each patient. During follow-up, we collected information about the arrival and departure times, activities patients were involved in before and after the clinic, the monetary value of these activities, their views on waiting time and the length of time they spent at the clinic. To obtain the time participants spent at the clinics, we obtained the difference between arrival and departure times. To get the value of the work participants were involved in on their clinic appointment days, we used replacement cost method, which measures the value of work by reference to what it might cost to hire someone to do the work [21]. In this regard, we asked them the nature of the work they did before and after their clinic appointment and how much they would pay if they were to hire someone to do the work for them. Thus, we obtained and reported the mean duration the participant spent at the clinic and the value of work reported for all the visits. Ethical approval for this study was obtained from Kenya Medical Research Institute's Ethical Review Committee under protocol review number SSC 2102.

### Data analyses

The data were entered in CSPro 5.0.3 (Maryland, U.S. Census Bureau) with built-in data quality control checks and exported into SPSS version 18 (Chicago, SPSS, Inc.) for further data cleaning and analyses. We used both descriptive and inference statistics for analyses. Specifically, we used counts and percentages to describe our data and chi-square (*χ*^2^) test to test whether differences existed between patients in the two study arms in respect to socio-demographic and treatment characteristics. Due to our data not meeting normality test, we used a non-parametric Mann–Whitney *U* test for our key endpoints which tested for the differences between the median waiting time and the value of work done before and after clinic for participants in the two study arms.

For our key endpoints, we performed two separate analyses. We first performed a complete case analysis that included only patients with complete data on key endpoints at the end of the study. We then conducted an intention-to-treat analysis as sensitivity analysis on imputed data whose missing values on our key endpoint had been replaced through a process of multiple imputation. We carried out expectation–maximization (EM) test to establish whether or not data were missing completely at random (*χ*^2^=230.6, DF=191, *p=*0.03). The significant *p*-value results meant that we could only use data missing not at random assumptions during multiple imputation. Running multiple imputations, as opposed to single imputations, accounts for the statistical uncertainty in the imputations. The multiple imputation process used baseline covariate information (age, sex, area of residence, means of transport to care clinic, reported time taken at clinic and value of work on clinic days) to run five imputations. The resultant data set allowed us to use data from all 354 patients randomized. We then performed a sensitivity analysis to determine the effect of missing data on our outcome measures in the two study arms.

## Results

### Enrolment and loss to follow-up

Figure 1 shows the CONSORT flow diagram for participants in the course of the study. Overall, 354 participants were enrolled into the study; 177 in each arm. A total of 13 participants were lost to follow-up in the intervention arm leaving 161 (91.0%) in the analysis. Of the thirteen, five transferred out of the programme, another five were lost to follow-up in the programme and three withdrew from the study citing lack of time to respond to a brief follow-up questionnaire. In the control arm, 29 participants were lost to follow-up leaving 148 (83.6%) for final analysis. Out of the 29, three transferred out of the programme, 16 were lost to follow-up from the programme and 10 withdrew from the study; five citing lack of time to respond to study follow-up questionnaire, four reporting forgetting to report to the study office and one withdrawing due to undisclosed personal reasons.

### Socio-demographic and economic characteristics

Overall, the study population consisted of 61.6% female, 70.0% were married and 63.8% had only primary-level education. Participants were predominantly (94.2%) from the Luo ethnic group and most of them professed Christian faith (97.4%). Over half (53.2%) were below 40 years old with 66.4% having more than one child. Most of the patients (84.2%) were largely employed. There were no significant differences between intervention and control groups with respect to these attributes (Table 1).

**Table 1:** Socio-economic and demographic characteristics of HIV patients enrolled in a designated time patient appointment system

Variable^a^	All	Intervention	Control	*p*-value (Fisher's exact test)

	Freq (%)	Freq (%)	Freq (%)	
Gender (*n*=354)				
Male	136 (38.4)	73 (41.2)	63 (35.6)	0.32
Female	218 (61.6)	104 (58.8)	114 (64.4)	
Level education (*n*=130)				
Primary	83 (63.8)	44 (64.7)	39 (62.9)	0.86
Secondary	47 (36.2)	24 (35.3)	23 (37.1)	
Ethnic group (*n*=308)				
Luo	290 (94.2)	144 (94.1)	146 (94.2)	1.00
Other	18 (5.8)	9 (5.9)	9 (5.8)	
Religion (*n*=309)				
Christian	301 (97.4)	147 (97.4)	154 (97.5)	1.00
Muslim	8 (2.6)	4 (2.6)	4 (2.5)	
Age (*n*=252)				
Below age 40	134 (53.2)	62 (50.8)	72 (55.4)	0.53
Age 40 and above	118 (46.8)	60 (49.2)	58 (44.6)	
Marital status (*n*=293)				
Currently married	205 (70)	106 (73.6)	99 (66.4)	0.20
Single (never married, separated, divorced, widowed)	88 (30)	38 (26.4)	50 (33.6)	
Number of living children (*n*=214)				
One or none	72 (33.6)	37 (36.6)	35 (31)	0.39
More than one child	142 (66.4)	64 (63.4)	78 (69)	
Employment status (*n*=336)				
Employed	53 (15.8)	32 (18.6)	21 (12.8)	0.10
Unemployed	283 (84.5)	140 (81.4)	143 (87.2)	

Freq = frequency;

a Different number of participants (*n*) with data on variables because of missing data from HIV clinic database.

### Residential, distance to clinic and clinic attendance information

Two-thirds of the participants (65.8%) lived within Kisumu City where the HIV care clinic is located. Over half of the participants (55.9%) reported doing some work before leaving home for the clinic on their clinic visit days (Table 2). About 45% of the patients use a single means of transport to get to their care clinic. However, 31.4 and 23.7% use two and three different means of transport, respectively, to get to the health facility. The means of transport used by most participants was walking (56.8%), *matatu* (48.6%) and motorbike taxi (40.1%). Most of the participants reported that their means of transport was readily available (92.7%) and reliable (90.1%). Slightly over one-third reported to take more than one hour to reach the clinic. While 13% did not incur any financial cost on transport to clinics, 46% spent more than KShs. 100 (USD~1.2). At the clinic, over half (52.4%) reported spending four to five hours waiting to be seen on their clinic day. Most of the participants had been in care for two to four years. About 46% had CD4 cell count >500 copies/mm^2^, and 81.4% recorded increase in CD4 cell counts since enrolment (Table 2).

**Table 2:** Residential, distance to clinic and clinic attendance information of HIV patients enrolled in a designated time patient appointment system

Variable	All	intervention	Control	*p*-value

	Freq (%)	Freq (%)	Freq (%)	
Residential area				
Within Kisumu Town	233 (65.8)	111 (62.7)	122 (68.9)	0.26
Outside Kisumu Town	121 (34.2)	66 (37.3)	55 (31.1)	
Work done before visiting clinic				
Yes	198 (55.9)	104 (58.8)	94 (53.1)	0.33
No	156 (44.1)	73 (41.2)	83 (46.9)	
Number of means of transport to clinic				
1	159 (44.9)	75 (42.4)	84 (47.5)	0.44
2	111 (31.4)	61 (34.5)	50 (28.2)	
3	84 (23.7)	41 (23.2)	43 (24.3)	
Availability of means of transport used				
Rare	26 (7.3)	14 (7.9)	12 (6.8)	0.84
Available	328 (92.7)	163 (92.1)	165 (93.2)	
Reliability of means of transport used				
Unreliable	35 (9.9)	15 (8.5)	20 (11.3)	0.48
Reliable	319 (90.1)	162 (91.5)	157 (88.7)	
Time taken to reach the clinic				
Less than 60 minutes	231 (65.3)	113 (63.8)	118 (66.7)	0.65
More than 60 minutes	123 (34.7)	64 (36.2)	59 (33.3)	
Transport cost				
No financial cost	46 (13)	22 (12.4)	24 (13.6)	0.50
Up to KShs 100	145 (41)	68 (38.4)	77 (43.5)	
More than KShs 100	163 (46)	87 (49.2)	76 (42.9)	
Overall time taken at clinic				
Up to 3 hours	77 (21.8)	39 (22)	38 (21.6)	0.92
4–5 hours	185 (52.4)	91 (51.4)	94 (53.4)	
More than 5 hours	91 (25.8)	47 (26.6)	44 (25)	
Duration of time in care				
Less than two years	61 (19.6)	30 (19.6)	31 (19.6)	1.00
2–4 years	130 (41.8)	64 (41.8)	66 (41.8)	
Over four years	120 (38.6)	59 (38.6)	61 (38.6)	
Most recent CD4 cell count/mm^3^				
500 and less	156 (54.2)	83 (58.9)	73 (49.7)	0.12
More than 500	132 (45.8)	58 (41.1)	74 (50.3)	
Changes in CD4 since enrolment				
Declining CD4 cell count/mm^3^	53 (18.6)	21 (15)	31 (22.1)	0.13
Increasing CD4 cell count/mm^3^	232 (81.4)	119 (85)	113 (77.9)	

Freq = Frequency.

### Clinic arrival time for participants on intervention

**Figure 2:**
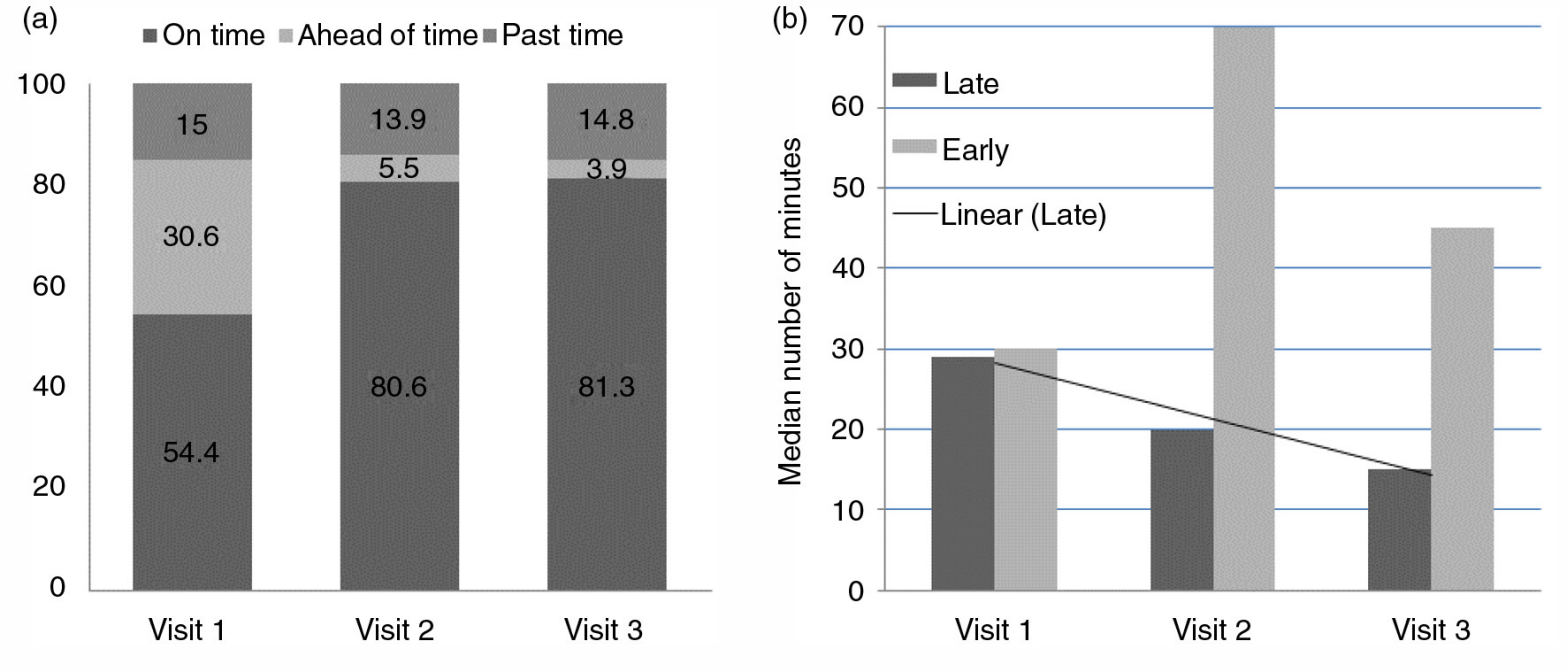
(a) Arrival status of participants in relation to their designated clinic time and (b) median number of minutes participants arrive on time or late.

Over a half of participants (54.4%) arrived on time for their scheduled clinic appointment on their first visit after randomization. This proportion increased to 80.6 and 81.3% on their second and third visits, respectively. The proportion of patients arriving early, on time and past time was slightly different after imputing the missing values for our outcome variable – arrival time at the clinic (see Supplementary file 1). On the other hand, 15.0% of the participants arrived past their scheduled time for their clinic appointment on their first visit post-randomization. The proportion arriving past their scheduled time did not seem to change much in their second (13.9%) and third (14.8%) visits (Figure 2a). About one-third of the participants (30.6%) arrived ahead of their scheduled appointment time in their first visit after randomization. This proportion decreased in each of the two subsequent visits. Participants arriving past their scheduled time on the first visit were late by a median of 29 minutes [interquartile range (IQR), 22–47] while those arriving past their scheduled time on the second visit came in within a median of 20 minutes (IQR, 15–50). The median number of minutes the participants were late decreased a further 15 (IQR, 10–38) in their third clinic visit (Figure 2b). Conversely, participants arriving ahead of time on their first visit after randomization came early by a median of 30 minutes (IQR, 10–75). This increased to a median of 70 minutes (IQR, 13–176) in their second visit and then dropped to 45 minutes (IQR, 28–108) in their third visit.

### Time savings and economic productivity

Table 3 shows the difference in time spent at the clinic and economic productivity between participants in the intervention and control arms. During visit 1 follow-up, intervention participants spent a median of 77 (IQR, 53–110) minutes at the clinic compared to a median of 223 (IQR, 191–272) minutes for participants in the control arm (*p*<0.01; Table 3). Similarly, intervention participants spent much less time at the clinic in visits 2 and 3 compared to control participants (*p*<0.01). When we evaluated the value of work, participants were engaged in before and after their clinic visits, we found no significant differences between intervention and control arm in visits 1 and 2 (Table 3). However, intervention participants were more productively engaged before and after clinic during visit 3 compared to control participants. Overall, the cumulative value of work done by intervention participants in all the three visits was significantly higher than the value of work control participants were engaged in before and after their clinic visits (USD 10.5 versus 8.3; *p*=0.02). Even after imputation for missing data in primary outcome variable, there was no relevant influence on the results (see Supplementary file 2).

**Table 3:** Time spent at clinic and value of work done on clinic visit day

Variable	Intervention	Control	*p*^a^

	Median (IQR)	Median (IQR)	
Time spent at care clinic (minutes)			
Visit 1	77 (53–110)	223 (191–272)	<0.01
Visit 2	49 (38–75)	181 (144–208)	<0.01
Visit 3	55 (45–79)	180 (136–219)	<0.01
Average time in three visits	65 (52–87)	197 (173–225)	<0.01
Value of work done before and after clinic (USD)			
Visit 1	3.3 (1.1–5.5)	2.8 (1.7–4.4)	0.31
Visit 2	3.3 (1.1–5.5)	2.8 (1.7–5.0)	0.42
Visit 3	3.3 (1.1–5.5)	2.3 (1.1–4.4)	<0.01
Cumulative amount in three visits	10.5 (6.0–16.8)	8.3 (5.5–12.9)	0.02

a *p*-value from Mann–Whitney *U* test statistics.

## Discussion

In this study that assessed the feasibility and economic impact of instituting a time designated appointment system in a busy HIV care clinic, we found that most patients were able to arrive on time for their scheduled appointments. As expected, patients in the intervention arm spent significantly less time at the clinic, on average slightly over two hours less time, and were more productively engaged in their livelihood activities on the day of their clinic visit. The proportion of participants arriving on time increased with each of the three visits but those arriving past scheduled time remained approximately the same.

To our knowledge, this is the first study in sub-Saharan Africa that has attempted to assess whether HIV patients can keep their appointment times at care clinics to ensure that they are only available at the clinic when required. This intervention was designed to limit waiting time and improve efficiency, which has been associated with non-adherence and attrition from care with inevitable ART interruptions, viral rebound and increased likelihood of drug resistance [14, 15, 22]. Many studies have mostly focused on patient flow analyses to detect inefficiencies and time wastage to institute corrective measures aimed at reducing overall time at the clinic [23–25]. These studies found workflow inefficiencies in the system that could be addressed to enhance patient flow [8, 26, 27]. At high volume clinics, enhancing workflow alone will not address a patient's time at the clinic if everyone arrives at the same time usually in the early morning. Time spent attending clinical appointments places strain on patients and their families by competing with socio-cultural and income generating activities [14]. Our results show that patients attending HIV clinics are able to arrive at scheduled times contrary to earlier scepticisms about African's ability to keep time. The argument for scepticism was that Africans lacked a requisite concept of time to adhere to timing of important events [28]. In fact, adherence has been found to be better in Africa than North America [29]. Since most patients enrolled in HIV care understand that ARV treatment is an important lifetime commitment, keeping appointments is a high priority for patients and time savings allow them more time to engage in other important daily activities [30].

In many HIV care programmes, it is not uncommon to find patients arriving as early as 4 am to have a better chance of being served among the first people when clinics reopen at 8 am [9]. Such patients forego many important routine tasks which, if not considered in HIV care programming, may eventually affect their lives and even retention in care. Time spent at the clinic in this study seems to have had differential effects on lost to follow-up between our intervention and control groups. People with a higher value of time might have been more likely to go to a scheduled appointment while at the same time, and people with a high value of time in the control group might have absconded their clinic because of the anticipated waiting time. One way of being considerate of the patients’ competing life tasks is to implement a designated time appointment system in HIV care clinics that streamlines clinic attendance to reduce waiting time and allow patients to plan and engage in their routine socio-economic activities. We have demonstrated that patients on designated time appointment spent significantly reduced time at the care clinics and are productively engaged before and after their clinic attendance compared to patients on standard scheduled drop-ins. In many instances, the value of patients’ time is generally reflected as their wages. For many people in Kenya who depend on casual employment and daily wages, anything that competes for time with work that supports their families has impact on the quality of their lives. This has widely been cited as one of the reasons for poor retention in care in HIV care programmes as people prioritize their sources of livelihood over clinic attendance [14, 31]. Poor retention in care undermines clinical outcomes and eventually the entire national HIV programmes. Thus, as noted by Decroo *et al*., models of HIV care need to adapt effective ways to support continued scale-up of ART and retain millions in care [32]. This can be accomplished through implementing models such as designated time appointment systems that streamline clinic attendance; making it efficient and friendly.

We noted several limitations in this study. First, this study was conducted in a single high volume HIV clinic that served both intervention and control participants by the same clinical staff. While we ensured that each day's workload was shared equally among the clinic staff so that intervention participants are not favoured by being fast-tracked over control participants, it is possible that this might have happened. This is especially the case when there were differentials in daily staff numbers at the care clinic. In addition, the study arm assignment was unblinded to both patients and staff, which could have altered their behaviour depending on the arm assigned. Second, we conducted this study in a large busy urban HIV care clinic, which might prove very different operationally in regard to several factors including staffing dynamics compared to more rural or less busy clinics. Third, this urban clinic serves patients within urban and peri-urban areas with better transport and road networks, which might have aided the participants to arrive on time. Thus, the results from this study may not be generalizable to rural clinics. Four, we only focused on the feasibility of patients keeping time and utilizing the time saved at the clinic without considering clinic operations, staffing and staff attitudes that might affect the designated time appointment system. We also did not consider the benefits that might be accrued by the staff and clinic management as a result of this system. The designated time appointment system might have both positive and negative effects on the clinic operation. For instance, unless the clinic has operational and organizational capacity to adhere to patients’ scheduled time, the system may result in overflows and eventually be rendered ineffective. Five, this study had a follow-up period of nine months or three follow-up visits. This duration might not have been sufficient to demonstrate sustainability and durability of the intervention. Lastly, by enrolling patients at the clinic, the sample included more adherent patients with lower loss to follow-up rates than a sample of newly diagnosed patients, or any sample that included all the patients at the same starting point. The result would be generalized to a stable patient population rather than one of newly diagnosed patients. Additionally, the study had a significant number of participants who were lost to follow-up either because they were transferred out or were lost to follow-up from the clinic. Since the design of this study was explanatory rather than pragmatic, we first conducted complete case analysis before sensitivity analysis within the intention-to-treat analysis framework.

## Conclusions

Although some patients seemed apprehensive about the system as depicted from the slightly lower numbers arriving on time in their first follow-up visit post randomization, they adjusted to arrive on time for their subsequent visits. Additionally, the intervention accrued time savings, and patients productively used the time saved at the clinic. Thus, implementation of a designated time appointment system in HIV care clinics in this setting is feasible and provides substantial time savings for patients and was associated with greater economic productivity for HIV patients at a busy HIV care and treatment clinic. Further research is required to determine if a designated time appointment system is scalable, and functions well in other settings with integrated care or less busy clinics, including within rural communities who may have different transport or access challenges. Additionally, since the current study was focused on the patients, further research should explore how time-based appointments can affect the health system including provider performance and satisfaction.

## Supplementary Material

The feasibility, time savings and economic impact of a designated time appointment system at a busy HIV care clinic in Kenya: a randomized controlled trial

The feasibility, time savings and economic impact of a designated time appointment system at a busy HIV care clinic in Kenya: a randomized controlled trial
