# Supplementary material for: The feasibility, time savings and economic impact of a designated time appointment system at a busy HIV care clinic in Kenya: a randomized controlled trial
Source: J Int AIDS Soc. 2015 Jul 9;18(1):19876. doi: 10.7448/IAS.18.1.19876 (PMC4499059; doi:10.7448/IAS.18.1.19876)
Supplement: The feasibility, time savings and economic impact of a designated time appointment system at a busy HIV care clinic in Kenya: a randomized controlled trial [file JIAS-18-19876-s001.pdf]

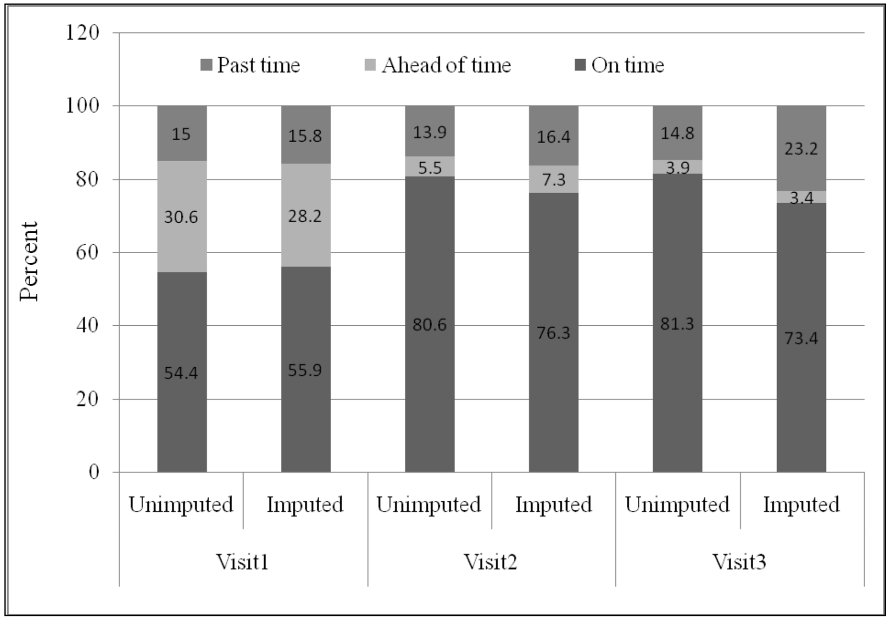


Participant arrival status in relation to clinic designated time compared between imputed and unimputed data

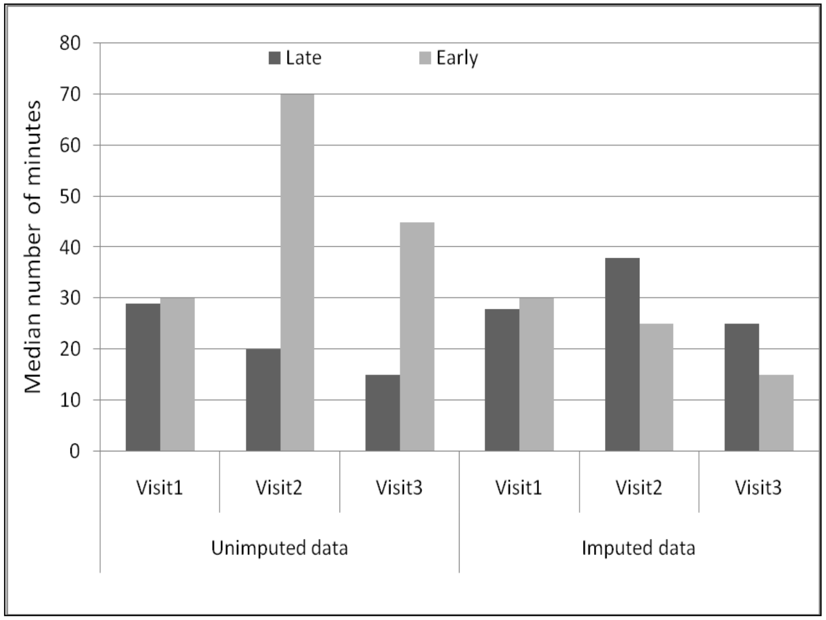


Median number of minutes participants arrived late or early compared between imputed and unimputed data
