# Supplementary material for: The feasibility, time savings and economic impact of a designated time appointment system at a busy HIV care clinic in Kenya: a randomized controlled trial
Source: J Int AIDS Soc. 2015 Jul 9;18(1):19876. doi: 10.7448/IAS.18.1.19876 (PMC4499059; doi:10.7448/IAS.18.1.19876)
Supplement: The feasibility, time savings and economic impact of a designated time appointment system at a busy HIV care clinic in Kenya: a randomized controlled trial [file JIAS-18-19876-s002.pdf]

Influence of multiple imputation on primary endpoint results

Variable	Unimputed data			Imputed data		
	Intervention [median(IQR)]	Control [median(IQR)]	p-value	Intervention [median(IQR)]	Control [median(IQR)]	p-value
Time spent at care clinic (minutes)						
Visit 1	72(51-103)	223(191-272)	<0.001	77(53-110)	228(191-272)	<0.001
Visit2	49(38-75)	181(144-208)	<0.001	51(38-77)	181(143-215)	<0.001
Visit2	55(45-79)	180(136-219)	<0.001	57(45-90)	185(148-223)	<0.001
Average time in 3 visits	65(52-87)	197(173-225)	<0.001	68(54-91)	197(175-225)	<0.001
Value of work done before and after clinic (USD)						
Visit 1	3.3(1.1-5.5)	2.8(1.7-4.4)	0.313	2.8(1.7-4.4)	3.3(1.1-5.5)	0.197
Visit2	3.3(1.1-5.5)	2.8(1.7-5.0)	0.425	2.8(1.6-5.0)	3.3(1.1-5.5)	0.399
Visit2	3.3(1.1-5.5)	2.3(1.1-4.4)	0.009	2.2(0.8-4.4)	3.3(1.1-5.5)	0.003
Cumulative amount in 3 visits	10.5(6.0-16.8)	8.3(5.5-12.9)	0.017	8.3(5.4-13.3)	10.5(6.1-17.2)	0.004

*p-value from Mann Whitney U test statistics
